# Reasons for Day-of-Surgery Cancellation of Elective Surgical Procedures During the COVID-19 Pandemic Compared to Pre- and Post-COVID-19 Periods

**DOI:** 10.7759/cureus.74205

**Published:** 2024-11-22

**Authors:** Paweł Topolewski, Dariusz Szplit, Jarek Kobiela, Dariusz Łaski, Pieter Stepaniak, Tomasz J Stefaniak

**Affiliations:** 1 Division Of Quality in Healthcare, Medical University of Gdańsk, Gdańsk, POL; 2 Department of Innovation and Clinical Processes Analysis, University Clinical Center of Medical University of Gdańsk, Gdańsk, POL; 3 Division of Quality in Healthcare, Medical University of Gdańsk, Gdańsk, POL; 4 Department of Surgical Oncology, Transplant Surgery and General Surgery, Medical University of Gdańsk, Gdańsk, POL

**Keywords:** covid-19, crisis management, medical logistics, operating rooms, operating theatre

## Abstract

Introduction: With the emergence of the global pandemic caused by SARS-CoV-2, health service providers were put to the test. The utilization of operating theatres is one of the prime indicators of the logistic and organizational efficacy of a hospital. We performed an analysis evaluating the impact of the COVID-19 pandemic on the fluidity of providing surgical care to a patient with severe comorbidities and the organizational efficacy of the operating theater in a university hospital in northern Poland.

Methods: Casemix between the periods was tested and Fisher’s test was used to examine the significance of the association between (i) patient, (ii) workup, (iii) resource/facility, and (iv) bed-related causes of day-of-surgery cancellations in pre- and post-COVID-19 pandemic periods and early- and medium-COVID-19 pandemic periods.

Results: In the study period, a total of 1,935 elective surgeries were booked, 2,219 elective and emergency surgeries were performed, and there were 203 (10.5%) cancellations. Patient-related cancellations increased significantly during the COVID-19 pandemic, accounting for 18.8% of cancellations, compared to 5.1% in pre- and post-pandemic periods (*p* = 0.0115). Resource/facility-related reasons remained the most frequent cause, contributing to 57.3% of cancellations during the pandemic.

Conclusions: Patient-related causes were the most frequent reasons in the COVID-19 pandemic period. Furthermore, patient-related reasons may have contributed to a drop in demand for surgical care and a nonsignificant change in the correlation between demand and supply for surgical care. The further effects of the COVID-19 pandemic on surgical department work are yet to be observed and should be reported to find a solution for growing societies’ need for surgical care and find tools to minimize the “damage” caused by the next crisis in global healthcare.

## Introduction

With the emergence of the COVID-19 pandemic caused by SARS-CoV-2 [1], health service providers were put to the test. Restrictions implemented by local governments caused disruptions in the regular work of almost every health service organization. The healthcare system was focused mainly on treating patients with COVID-19 and, at the same time, protecting their own personnel. The rapid increase in the inflow of more COVID-19-related diseases resulted in complex organizational challenges for both management and professionals. Operating rooms (ORs) are a scarce resource that is both relatively high labor and capital-intensive. Efficient use of the operating theater is therefore not only a challenge during normal periods but also during healthcare crises like the COVID-19 period. Efficient use of OR capacity is equal to minimizing OR inefficiency [2].

If patients scheduled for the OR are canceled and these gaps in the OR schedule cannot be filled with another surgery, then the OR inefficiency increases. In other words, OR efficiency decreases. As in many hospitals worldwide, we observed an increase in OR cancelations during the COVID-19 period.

In this study, we analyze the effect of the COVID-19 period on the efficient use of operating rooms in an academic hospital compared with the pre- and post-COVID-19 periods. The aim of this study was to perform an analysis to evaluate the impact of the COVID-19 pandemic on the cancellation of planned surgical procedures in operating theater complexes in university hospitals. This study is the first to examine the impact of COVID-19 on elective surgical procedure cancellations and operating theater utilization. By analyzing the reasons for day-of-surgery cancellations and proposing strategies to maintain critical services, we provide practical insights for managing surgical care during future healthcare crises.

## Materials and methods

Study design and setting

The study was conducted at the University Clinical Center of the Medical University of Gdańsk, Poland, with 26 ORs. OR capacity is assigned to 14 surgical specialties. We conducted a retrospective analysis evaluating (i) the number of planned surgeries and (ii) the number, type, and reasons for surgery cancellation.

During the COVID-19 pandemic period, our center was not predefined as a hospital providing care to COVID-19 patients. Nevertheless, our hospital developed 120 beds for COVID-19 patients, mostly identified and transferred from other wards. Those patients were initially hospitalized due to non-COVID specialistic reasons (oncology, hematology, etc.) and then revealed COVID-19 infection. Another group included patients suffering from specialistic health problems who developed COVID-19 at home and were transferred to our hospital due to a lack of expertise within the COVID-19-designated hospitals. The conception of prehospitalization and COVID-19 testing prior to admitting patients to a hospital was then implemented. However, all the COVID-19 positive test results that have been used as a reason for elective surgery cancellation were an effect of hospital-acquired infection or false-negative tests performed before the hospitalization. We also concentrated our efforts on maintaining an adequate number of oncologic and transplant procedures, as those groups of patients could be mostly affected by any drop in the number of planned surgeries.

The analyses, due to the need for comparison, were performed in four six-week time periods: pre-COVID-19-pandemic (June 3, 2019-July 15, 2019), early COVID-19 pandemic (March 2, 2020-April 10, 2020), medium COVID-19 pandemic (March 2, 2021-April 12, 2021), and post-COVID-19-pandemic (February 28, 2022-April 10, 2022). The study periods were selected as six-week intervals, avoiding interruptions from national holidays and other events, following the standard methodology for analyzing OR performance. (The pre-COVID-19 period included June 2019 instead of March 2019 due to a lack of complete data regarding operating theater utilization in March 2019.) All patients were admitted to the surgical unit and had their hospital bed in the relevant surgical department. For analytical purposes, five surgical units (Department of Plastic Surgery, Department of Maxillaro-Facial Surgery, Department of Otolaryngology, Department of Thoracic Surgery, and Department of Urology) were chosen for the analyses due to having a nonsignificantly different casemix among the periods in the study. The performance of the five departments indicates that 24.83% of all surgeries were performed at our OR complex.

All surgeries (elective and emergency) performed in the period were included in the study. The inclusion and comparison criteria are described in Table 1.

**Table 1:** PICO criteria used in the study. PICO: Patients, Intervention, Comparison, Outcomes

PICO	Description
P	Patients who had planned surgeries canceled in two months of operating theater complex utilization.
I	Categorical reasons for surgery cancellation.
C	Patients who had planned surgeries canceled in two months pre-COVID-19 pandemic and post-COVID-19 pandemic.
O	Influence of the COVID-19 pandemic on the number of and reasons for planned surgery cancellations and consequent insufficiency of healthcare resources in the post-COVID-19 period.

Data collection, outcome variables, and definitions

Data collection was performed based on an analysis of cross-sectional analytic reports provided daily by the personnel of the operating theatre complex. These data include the number and type of surgeries booked on the operating theatre complex, the number and type of surgeries performed in the operating theatre, and the number, type, reasons for surgery cancellation, and time of patient entering and leaving the operating theatre. Data were reported daily and synthesized weekly using the Airtable platform (San Francisco, California, United States).

Reasons for elective surgery cancellations were categorized according to the methodology proposed by Kaddoum et al. [3]. Categorical reasons for elective surgery cancellations are presented in Table 2.

**Table 2:** Categorical reasons for elective surgery cancellations. PCR: polymerase chain reaction

Category	Reason
Patient	Patient did not report to the hospital
	Patient resigned from the surgery
	Surgery was performed at another hospital
Workup/Medical condition	Change in medical status (general state change, infection, death)
	Change in therapeutic plan when surgery was already scheduled
	Necessity for intensified diagnostics
	Scheduled surgery performed sooner as an emergency
	Preoperative instructions not followed properly
Admission	Patient did not consent for the surgery before premedication
	No court’s agreement for the surgery
	Patient uninsured
	No COVID-19 PCR test performed when admitted to a hospital
Bed	No hospital beds available at postoperative intensive care unit
	No hospital beds available at surgical unit
Resource/facility	Surgery did not fit in the booking time
	Surgery canceled due to emergency or transplantation
	Surgery performed at surgical ward
	Surgery postponed
	Reason for cancellation not reported by the staff
Surgeon	No surgeon available
COVID-19	COVID (+)

Statistical analysis

Upon concluding data collection, the results were synthesized and analyzed in tables and visual graphs using Microsoft Excel 2022 (Microsoft Corporation, Redmond, Washington, United States). Casemix between the periods was tested by using the Herfindahl index, and the differences between the Herfindahl index among the periods were tested using the modified McKay’s confidence interval. The confidence level (Cl) was set at 95%. Fisher’s test was used to examine the significance of the association between (i) patient, (ii) workup, (iii) resource/facility, and (iv) bed-related causes of day-of-surgery cancellations in pre- and post-COVID-19 pandemic periods and early- and medium-COVID-19 pandemic periods. P values <0.05 were considered significant. Analyses were computed using GraphPad Prism Version 9.4.1 (Released 2022; Insightful Science, LLC, Boston, Massachusetts, United States).

## Results

In the analysis periods, 1935 elective surgeries were booked at the operating theater complex, 2219 elective and emergency surgeries were performed, and 203 were canceled for various reasons. The number of elective surgeries performed, all surgeries performed, and number and percentage of elective surgeries canceled are presented in Table 3. These data are with regard to departments included in the analysis.

**Table 3:** Summary of surgeries booked, performed, and canceled at the operating theater complex.

Period	Elective surgeries performed, n	All surgeries performed, n	Number of elective surgeries canceled	Percentage of elective surgeries canceled
COVID-19 pandemic period (six weeks periods)	971	1274	128	11.65%
2020 (March 2, 2020-April 10, 2020)	444	604	80	15.27%
2021 (March 2, 2021-April 12, 2021)	527	670	48	8.35%
Pre- and post-COVID-19 pandemic period (six weeks periods)	761	945	75	8.97%
2019 (June 3, 2019-July 15, 2019)	343	431	39	10.21%
2022 (February 28, 2022-April 10, 2022)	418	514	36	7.93%

The Herfindahl index was calculated for the four time periods. The Herfindahl index differences with 95% confidence intervals are presented in Table 3. The Herfindahl index was utilized to confirm that the surgical casemix remained consistent across all study periods, thereby minimizing potential bias. For all differences between time periods, the casemix did not differ significantly.

**Table 4:** Herfindahl-Index differences and confidence intervals.

Period analyzed	Herfindahl-Index difference value	Confidence interval (95% Cl)
2019-2020	0.021	-0.097-0.054
2020-2021	0.017	-0.063-0.097
2021-2022	0.014	-0.065-0.094

**Figure 1:**
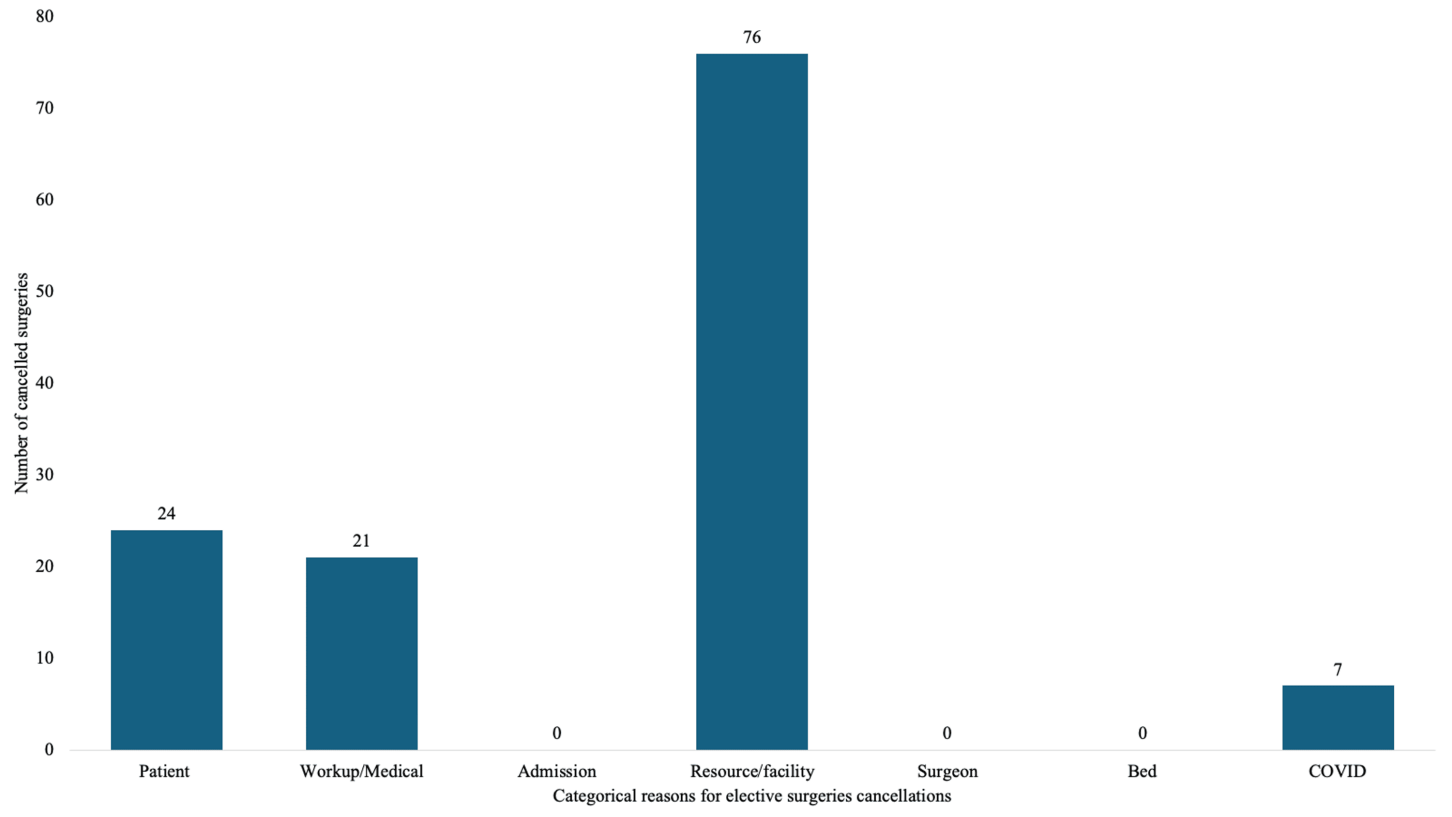
Number of elective surgery cancellations in the COVID-19 pandemic period cumulated distributed according to reasons (N=128)

In the COVID-19 pandemic periods, 128 elective surgeries were canceled for categorical reasons: patient-related (n=24, 18.75%), workup-related (n=21, 16.41%), resource/facility-related (n=76, 59.38%), and COVID-19 positive results (n=7, 5.47%). There was no admission, surgeon-related, or bed-related causes of elective surgery cancellation. The distribution of the categorical reasons for elective surgery cancellation in this period is presented in Figure 1.

**Figure 2:**
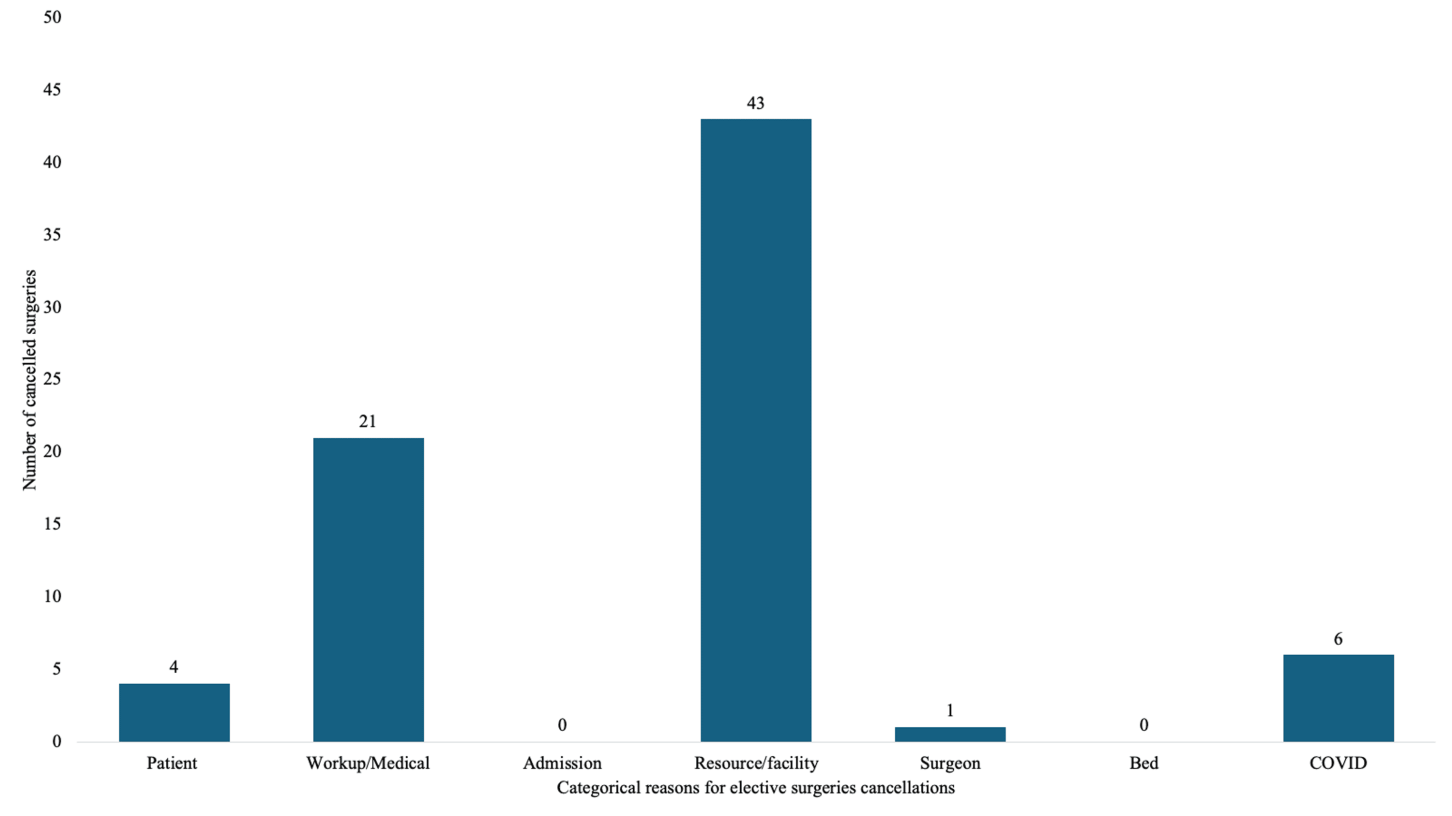
Number of elective surgery cancellations in pre- and post-COVID-19 pandemic periods cumulated distributed according to reasons (N=75)

In the pre- and post-COVID-19 pandemic periods, 75 elective surgeries were canceled for categorical reasons: patient-related (n=4, 5.33%), workup-related (n=21, 28.0%), resource/facility-related (n=43, 57.33%), surgeon-related (n=1, 1.33%), and COVID-19 positive results (n=6, 8.0%). There were no admissions or bed-related causes of elective surgery cancellation. The distribution of the categorical reasons for elective surgery cancellation in this period is presented in Figure 2.

In the statistical analysis, the following data in the pre- and post-COVID-19 pandemic period vs the COVID-19 pandemic period were compared: number and percentage of elective surgeries performed vs emergency surgeries performed, number of elective surgeries performed vs elective surgeries canceled, number and percentage of patient-related, work-up related, resource/facility-related, and bed-related reasons for elective surgery cancellations. The results are presented in Tables 5-7.

**Table 5:** Results of Fisher tests of the number and percentage of elective and emergency surgeries performed in the study periods P-values <0.05 were considered significant.

Study periods	Elective surgeries performed, n (%)	Emergency surgeries performed, n (%)	p value
Pre- and post-COVID-19 pandemic period (n=1146)	971 (84.73%)	175 (15.27%)	0.0813
COVID-19 pandemic period (n=870)	761 (87.47%)	109 (12.53%)	

**Table 6:** Results of Fisher tests of the number and percentage of elective surgeries performed and canceled P-values <0.05 were considered significant.

Study periods	Elective surgeries performed, n (%)	Elective surgeries canceled, n (%)	p value
Pre- and post-COVID-19 pandemic period (n=1146)	971 (88.35%)	128 (11.65%)	0.0612
COVID-19 pandemic period (n=870)	761 (91.03%)	75 (8.97%)	

**Table 7:** Results of Fisher tests of the number and percentage of categorical reasons for elective surgery cancellation P-values <0.05 were considered significant.

Categories	Pre- and post-COVID-19 pandemic period (n=1146), n (%)	COVID-19 pandemic period (n=870), n (%)	p value
Analysis of patient-related reasons for elective surgery cancellation			
Patient-related reasons for surgery cancellation	4 (5.13%)	24 (18.75%)	0.0115
Reasons other than patient-related reasons	74 (94.87%)	104 (82.25%)	
Analysis of workup-related reasons for elective surgery cancellation			
Workup-related reasons for surgery cancellation	21 (28.0%)	21 (16.41%)	0.0717
Reasons other than workup-related reasons	54 (72.0%)	107 (83.59%)	
Analysis of resource/facility-related reasons for elective surgery cancellation			
Resource/facility-related reasons for surgery cancellation	76 (59.38%)	52 (57.33%)	0.8827
Reasons other than resource/facility-related reasons	43 (40.63%)	32 (42.67%)	
Analysis of COVID-related reasons for elective surgery cancellation			
COVID-related reasons for surgery cancellation	7 (5.47%)	6 (8.0%)	0.5563
Reasons other than COVID-related reasons	121 (94.53%)	69 (92.0%)	

Statistical significance was achieved only in patient-related reasons for elective surgery cancellations. No statistically significant difference was found in elective surgeries performed vs emergency surgeries performed, elective surgeries performed vs elective surgeries canceled, and workup, resource/facility-related, and bed-related reasons for elective surgery cancellations.

## Discussion

Elective surgery cancellation is a major healthcare concern directly affecting patients’ physical and mental health [3]. Reported incidences of elective surgery cancellation range from 1% to 23% [4-6], with a recommended rate of less than 5% [7]. We found in our hospital that the overall percentages of canceled elective surgeries in all elective surgeries booked were 11.65% in the pre- and post-COVID-19 pandemic periods and 8.97% in the COVID-19 pandemic period.

The main categorical reasons for cancellations of elective surgeries were common in the two study periods and were resource/facility-related and workup-related. In the COVID-19 pandemic period, patient-related reasons for elective surgery cancellation were significantly higher. The number of booked, performed elective and emergency surgeries, and canceled elective surgeries was higher in the pre- and post-COVID-19 pandemic periods than in the COVID-19 pandemic period, and a growing trend in the included four periods of the study in these numbers was observed.

Our study found that with the growing overall number of performed surgeries, the ratio of performed emergency surgeries and canceled elective surgeries did not significantly differ in the pandemic period of the study. This indicates that the overall proportion between the demand and supply for surgical care, including emergency surgical care, was not imbalanced during the COVID-19 pandemic period, despite a drop in supplying surgical care, as some of the hospitals were transformed for COVID-19-only health centers.

No significant difference in resource/facility-related, workup-related, and bed-related reasons for elective surgery cancellation is a good argument for the hypothesis that the COVID-19 pandemic did not cause an imbalance between demand and supply for surgical care during the COVID-19 pandemic period. However, there was a significant difference in patient-related reasons for elective surgery cancellation. From our results, we cannot conclude what influenced patients’ decisions; however, there are numerous explanations, including fear of reporting to a hospital despite all the safety measures and many others. The possibility of patients avoiding hospital visits due to fear or misinformation could be a significant factor contributing to changes in patient-related reasons for elective surgery cancellations. Widespread campaigns encouraging people to stay home may have further influenced patients' decisions to undergo surgery. Reports in the literature highlight the anxiety induced by pandemics, which has led to delays in oncological treatment [8]. The impact of global pandemics on patient decisions has been particularly emphasized for cancer patients, with consistent findings underscoring the need to prioritize their physical and mental health during large-scale disruptions to cancer care [9]. While the sociological explanation of this phenomenon is not the primary focus of this research, these conclusions should be acknowledged and incorporated into future recommendations. We can also reflect that neither the everyday hospital work at the surgical unit nor managing the resources of the OR were influenced by the COVID-19 pandemic. Additionally, COVID-19 infection was not a significant factor influencing elective surgery cancellation. However, the impact of COVID-19 infection as a reason for elective surgery cancellation was minimized by the implementation of prehospitalization in our center.

Patients scheduled for elective surgery were obligated to arrive at a hospital a day earlier or perform a COVID-19 polymerase chain reaction (PCR) test. Then, they returned to the place of residence and remained self-isolated until the test result arrived. If the test result was negative, the patient could be admitted to a hospital. Therefore, all day-of-surgery cancellation due to COVID-19 infections in hospitalized patients (six in the COVID-19 pandemic period and seven in the pre- and post-COVID-19 pandemic periods) was an outcome of false-negative tests or infection acquired in a hospital. Hence, the number presented in this study does not reflect the real burden of COVID-19 infections on the organization of operation schedules during the pandemic months. Nevertheless, we presented it in that way, as we believe it reflects the real impact the unspotted infections had on the logistics of our OR complex. If the test was positive, the patient was not hospitalized and was not included in the final surgical list. If needed, he or she was referred to a hospital dedicated to providing healthcare to COVID-19-positive patients or was obligated to self-isolate in the place of residence until the infection was cured. These numbers prove that the concept of prehospitalization and the obligation to perform PCR tests before admitting a patient to a hospital were effective ways to protect hospitalized patients and healthcare providers. This was a crucial solution in dealing with the COVID-19 pandemic in our center, as most of the surgeries performed were oncological, and these patients were highlighted as a group with a higher risk of fatal infection [10,11].

Our results are not consistent with the other studies. Hunger et al. reported a decrease in elective (by 14.8%) and emergency (by 6.0%) surgeries in a study including 66 hospitals in Germany [12]. They did not report reasons for elective surgery cancellation but compared it to prediction models based on experts’ expectations. The actual decrease in the number of surgeries was lower than expected and was explained by the methodology of the prediction models and the strong seasonal impact of COVID-19 on surgical cancellation and immediate rescheduling. Therefore, we cannot fully relate to the results of Hunger et al.'s study, as we included only our center’s experience, and the design differed among the studies. In our center, a drop in the number of elective (by 21.63%) and emergency (by 37.71%) surgeries was observed. The difference could be explained by implementing prehospitalization and redirecting COVID-19-positive patients to hospitals assigned to provide surgical care to those patients. Mehta et al. underlined the issue of physician shortages with a special emphasis on surgical specialists and recommended “damage control”, such as the conception of prehospitalization [13], which was implemented in our center. In our center, due to the strict policy on PCR testing among physicians with the smallest COVID-19-like symptoms, prehospitalization PCR for elective patients, and rotation of staff, when possible, the impact of COVID-19 on human resources was minimized.

Our findings may be generalized across all surgical specialties in terms of OR management and reducing day-of-surgery cancellations. We recommend that future healthcare crises caused by infectious diseases could be mitigated through the adoption of prehospitalization protocols (mandatory testing prior to admission) and strategies to alleviate patient anxiety while fostering a sense of safety in accessing healthcare services. To ensure optimal OR performance, particular attention should be directed towards efficient resource utilization, as this was the primary cause of day-of-surgery cancellations. Implementing standardized procedures and management protocols for various patient groups can further enhance OR efficiency and minimize workup-related cancellations. We suggest that these measures can be applied across all surgical fields, as they represent universal strategies for enhancing management and ensuring maximum efficiency during global healthcare crises.

The main limitations of our study are its retrospective nature and inability to minimize the bias arising from the selection of the periods included in the study. Due to a lack of reporting complete data regarding operating theater utilization in an equivalent six-week period in March 2019, we randomly chose six weeks (June 3, 2019-July 15, 2019), and therefore, the bias could not be minimized. We also found the development of new healthcare programs in our center as a factor introducing a possible bias in our results; however, we showed that the casemix was similar among the periods, and there was no significant difference between the types of surgical procedures performed in the periods.

## Conclusions

In our center, the number of canceled elective surgeries in the COVID-19 pandemic period did not change significantly. It was minimized by taking precautions to eliminate the influence of the COVID-19 pandemic on the cancellation of elective surgeries and well management of OR resources, despite the changing situation in global healthcare. Patient-related reasons were the only reasons that increased during the COVID-19 pandemic period. Furthermore, patient-related reasons may have contributed to a hidden drop in demand for surgical care and contributed to a nonsignificant change in the correlation between demand and supply for surgical care. The further effects of the COVID-19 pandemic on surgical department work are yet to be observed and should be reported to find a solution for growing societies’ need for surgical care and find tools to implement to minimize the “damage” caused by the next possible crisis in global healthcare.
